# Sandwich doctorate in Nursing: contributions to academic training and the Sustainable Development Goals

**DOI:** 10.1590/0034-7167-2024-0344

**Published:** 2025-06-20

**Authors:** Mariana Mendes, Denise Elvira Pires de Pires, Ianka Cristina Celuppi, Olga Maria Pimenta Lopes Ribeiro

**Affiliations:** I Universidade Federal de Santa Catarina. Florianópolis, Santa Catarina, Brazil; II Escola Superior de Enfermagem do Porto. Porto, Portugal

**Keywords:** Sustainable Development, International Educational Exchange, Health Postgraduate Programs, Technical Cooperation, Nursing, Desarrollo Sostenible, Intercambio Educacional Internacional, Programas de Posgrado en Salud, Cooperación Técnica, Enfermería

## Abstract

**Objectives:**

to report on the contributions of the sandwich doctorate to the training of Nursing PhDs, exploring and analyzing its impacts on promoting international collaboration between graduate programs and its contribution to Sustainable Development Goals 4, 5, and 10.

**Methods:**

this experience report describes a doctoral student’s journey abroad, supported by a scholarship from the National Council for Scientific and Technological Development (CNPq), at a Nursing School in Portugal, conducted between May and July 2023.

**Results:**

the results are presented in three sections: activities performed; contributions of the sandwich doctorate to achieving Goals 4, 5, and 10; difficulties encountered and strategies for overcoming them. The sandwich doctorate promotes the acquisition of new knowledge, professional and teaching development, and international partnerships.

**Final Considerations:**

international collaboration, facilitated by the sandwich doctorate, contributes to strengthening nursing as a health profession, which plays a highly relevant role in achieving the targets of the 2030 Agenda.

## RESUMEN

**Objetivos:**

relatar las contribuciones del doctorado sándwich para la formación de doctores en Enfermería, con exploración y análisis de los impactos en la promoción de la colaboración internacional entre programas de posgrado y su contribución a los Objetivos de Desarrollo Sostenible 4, 5 y 10.

**Métodos:**

relato de experiencia de una estudiante de doctorado en el exterior, realizado mediante la concesión de una beca por parte del Consejo Nacional de Desarrollo Científico y Tecnológico (CNPq), en una Escuela de Enfermería de Portugal, entre mayo y julio de 2023.

**Resultados:**

presentados en tres secciones: actividades realizadas; contribuciones del doctorado sándwich para el logro de los Objetivos 4, 5 y 10; dificultades encontradas y estrategias para superarlas. El doctorado sándwich promueve la adquisición de nuevos conocimientos, el desarrollo profesional y para la docencia, y las asociaciones internacionales.

**Consideraciones Finales:**

la colaboración internacional, facilitada por el doctorado sándwich, contribuye al fortalecimiento de la enfermería como profesión de salud, que tiene un papel altamente relevante en el logro de las metas de la Agenda 2030.

## INTRODUCTION

International collaboration among universities has proven essential for advancing knowledge in Nursing and strengthening academic relationships between educational institutions worldwide. In this context, the internationalization of initiatives between universities in Brazil and Portugal stands out, contributing to the mobility of graduate students and faculty through official agreements for scientific cooperation or through connections established within professional networks^(1)^.

International interinstitutional partnerships enable academic exchange and the production of knowledge across different contexts(1, 2). The sandwich PhD abroad is a Brazilian expression that refers to an academic training model in which the PhD student completes part of their Brazilian studies related to their dissertation at a foreign educational institution^(1, 3)^. In this regard, the sandwich PhD abroad emerges as a powerful tool to foster international collaboration and create new cooperation networks, while contributing to the development of critical and committed researchers focused on advancing science on a global scale.

Experiences of international cooperation contribute to enhanced professional training and performance, particularly by fostering the exchange of methodological and scientific knowledge and paving the way for evidence-based practice. Moreover, the international experience has significant cultural, social, and scientific repercussions for nurses^(3)^, making the sandwich doctorate abroad a strategic approach to developing these professionals’ skills and competencies on an international level^(1)^.

The sandwich PhD is part of broader international cooperation strategies. Through it, nurses gain access to diverse academic environments, pedagogical and technological resources, collaborate with international researchers, and engage with local cultures and systems. In addition to contributing to the professional and academic development of graduate students^(1, 2)^, these experiences enhance opportunities for accessing quality education and can serve as a powerful resource for reducing social and gender inequalities.

The benefits of this experience extend beyond the academic and professional development of nurses, contributing to the production of different forms of knowledge that directly impact society^(1, 3)^, thereby collaborating with the achievement of the Sustainable Development Goals (SDGs). In 2015, the United Nations (UN) approved 17 SDGs to be reached by 2030. These goals consist of guiding principles for global actions aimed at reducing poverty and inequalities among population groups, including health, while promoting peace and preserving life on the planet.

In July 2023, the UN launched the “Act Now” campaign in support of the SDGs, preceding the 78th United Nations General Assembly held in September of the same year. The campaign’s appeal arose due to stagnation in progress toward achieving the SDGs, given the “combined weight of climate disasters, conflicts, economic recession, and the lingering consequences of COVID-19”^(4)^.

The sandwich PhD enables students to gain a global perspective and engage with international research networks to create solutions for society’s macro-political problems, as outlined in the SDGs, including health. The experience detailed here took place in Portugal as part of the PhD studies of a Brazilian student. The doctoral research investigates technological innovations used by nurses in Primary Health Care (PHC) during the COVID-19 pandemic, aiming to understand their implementation processes and outcomes in the post-pandemic context, studying the phenomenon in both Portugal and Brazil.

Given the importance of promoting academic excellence in the pursuit of innovative and sustainable solutions that contribute to achieving the goals set out in the 2030 Agenda, the guiding question of this study is: How can a sandwich PhD program abroad contribute to advances in doctoral training and sustainable development?

This experience report highlights the contributions that the sandwich PhD can offer for the training and development of Nursing science, interpreting the results through the lens of global efforts to achieve a more sustainable world. In this study, the pursuit of SDG goals is considered, with a particular focus on three of them: 4 - Quality Education, 5 - Gender Equality, and 10 - Reduced Inequalities^(5)^.

## OBJECTIVES

To report the contributions of the sandwich PhD in Nursing, exploring and analyzing the impacts on promoting international collaboration between graduate programs and their contribution to SDGs 4, 5, and 10.

## METHODS

### Type of Study

This study adopts a qualitative, descriptive approach, characterized as an experience report, aimed at highlighting the contributions of the sandwich PhD abroad to the training of Nursing PhDs and to SDGs 4, 5, and 10.

### Theoretical Framework

For the conceptualization and discussion of the SDGs, the official UN Brazil website^(5)^ was utilized, along with a documental study. The documents intentionally chosen included official records, institutional reports, and scientific publications available online.

### Study Period and Context

The experience took place during a sandwich PhD abroad undertaken by a student of a Graduate Nursing Program from a Federal University in the Southern region of Brazil.

The setting for this experience was a Nursing School in Portugal. It involved the granting of sandwich PhD scholarships abroad (SWE) by the National Council for Scientific and Technological Development (CNPq in Portuguese) as a result of proposals submitted to the CNPq Call No. 26/2021 for Support of Scientific, Technological, and Innovation Research - Scholarships Abroad. The scholarship duration was three months, from May to July 2023.

The student’s acceptance at the host institution was formalized through contact between professors from Brazil and Portugal, facilitated by an existing international collaboration between them. This prior contact was fundamental to the development of the research project, including the study proposal, planning, and scheduling of activities to be carried out within the scope of the graduate program at the host institution.

### Data Organization

Data were organized into thematic sections to meet the study objectives. These sections include: a description of the activities carried out abroad and their relationship with PhD training knowledge production in Nursing, and contributions to the SDGs; the characterization of SDGs 4, 5, and 10 and their association with the sandwich PhD experience; and a discussion of challenges faced and strategies for overcoming them.

### Ethical Aspects

As this is an experience report, the study did not require approval from the Human Research Ethics Committee. However, the study has respected the international guidelines and Resolutions 466/12 and 510/16 of the Brazilian National Health Council, preserving anonymity and confidentiality for research purposes.

## RESULTS

The results of the experience are presented in three sections: activities carried out; contributions of the sandwich PhD to achieving SDGs 4, 5, and 10; challenges encountered and strategies for overcoming them.

### Activities Conducted During the Sandwich PhD Abroad

Chart 1 presents the activities conducted during the sandwich PhD, from May to July 2023, highlighting their contributions to PhD training, scientific advancement, and the achievement of the SDGs.

**Chart 1:** Activities Conducted, Contributions to PhD Training, Scientific Advancement, Research Partnerships, and Sustainable Development Goals Achievement

Activities performed	Contributions to PhD Training, Science, and Research Partnerships	Relationships with the SDGs
Speaker at the NursID Spring School event, sharing research conducted in Brazil on technological innovations in health and their relation to worker health, as well as theoretical perspectives of the PhD project.	Dissemination of knowledge and scientific exchange.	SDG 4 and 5
Participation in academic and scientific classes and events in Brazil and internationally, both online and in person.	Dissemination of research on technologies developed and used by nurses in Brazil.	SDG 4 and 10
Technical visits to local health units and a hospital specializing in COVID-19 care.	Identification of health service organization and management methods, as well as the nursing work process, with potential applications in other settings.	SDG 10
Data collection with PHC nurses on new technologies used during the COVID-19 pandemic.	Enriches the production of knowledge, expanding the understanding of the phenomenon between Brazil and Portugal.	SDG 4 and 5
Participation in the research groups Positive Professional Environments for Nursing Practice - Portugal, and Research Laboratory on Work, Ethics, Health and Nursing (PRAXIS) - Brazil.	Exchange and strengthening of research networks.	SDG 4 and 10
Collaboration in the production of scientific articles in partnership with international researchers.	Dissemination of scientific research results conducted by women and nursing knowledge.	SDG 4 and 5

### Contributions to Achieving the Sustainable Development Goals

**Figure 1:**
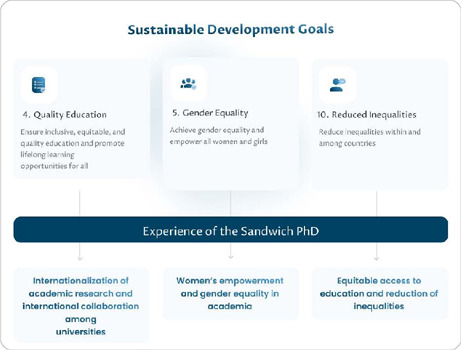
Sustainable Development Goals and the Sandwich PhD Abroad Experience

Figure 1 illustrates the selected SDGs—4, 5, and 10—and their connections with three key themes articulating the contributions of the sandwich PhD to their achievement.

### Internationalization of Academic Research and International Collaboration Among Universities

The exchange of knowledge among researchers of different nationalities enhances the quality and innovation of research. The sandwich PhD facilitates the sharing of diverse research approaches, which are essential for addressing the complex challenges faced by society today. In this way, the sandwich PhD not only promotes the internationalization of academic research and strengthens global collaboration among universities but also enhances the development and expertise of the professionals and researchers involved, aligning with SDG 4, which focuses on quality education worldwide.

Participation in academic and scientific events abroad provided opportunities to disseminate research conducted by Brazilian nurses and foster dialogue with international researchers. It also facilitated engagement with diverse research methodologies and the development of clinical studies and technologies applied to the work of Portuguese nurses (rehabilitation programs for surgical patients, technology for evaluating balance and gait in the elderly). This international experience inspires the development of more robust research aimed at improving health and nursing practices.

A particularly valuable aspect of the experience is the opportunity to collect data for the dissertation in an international context. Data collected in Brazil and Portugal contribute to producing a more consistent dissertation with greater global relevance, fostering critical discussions about the implementation of technologies, especially by PHC nurses. Through interviews with participating professionals, technical visits, and interactions with local health services, it was possible to learn about successful experiences of nurses using innovative technologies during the pandemic, with emphasis on videoconferencing platforms for synchronous communication with patients and the telemonitoring of patients with the disease.

The sandwich PhD also offers the opportunity to establish partnerships and cooperative relationships with leading researchers in their fields of study. The PhD student’s participation in an international research group focused on exploring positive environments for nursing practice expanded knowledge on the technologies used and their impact on health care and worker health.

The sandwich program strengthened skills in producing and submitting scientific articles to international journals, requiring proficiency in different languages, writing styles, and formatting of academic work. Developing these academic skills is also valuable for building networks and establishing future international partnerships.

### Women’s Empowerment and Gender Equality in Academia

The sandwich PhD plays a crucial role in promoting gender equality within the academic context. This is reflected in its ability to offer equal opportunities to women students and researchers, enabling them to access international academic environments, contribute through their research, and establish networks that strengthen their empowerment and positively influence gender representation in research and higher education. This process contributes to achieving SDG 5, which aims to eliminate gender inequalities and promote the equal participation of women in all sectors of society.

The experience of the PhD student reinforced the fundamental role of women in advancing nursing science and building academic knowledge, particularly in a predominantly female profession. Leading clinical studies, developing innovative technologies, coordinating research projects, and teaching in graduate programs are examples of activities led by female professors involved in supervising this program. The presence of women in high-level management positions, such as the two vice presidents of the School, also highlights opportunities for participation and leadership.

On the other hand, the experience facilitated the exchange of insights into the challenges faced by women seeking to advance in academia, such as managing the multiple dimensions of a woman’s life, which often encompass the roles of mother, spouse, and professional. This experience prompted reflections on the various roles assigned to women in society and how they can influence disparities in opportunities in both the job market and academic life.

### Equitable Access to Education and Reducing Inequalities

In the context of SDG 10, the exchange of PhD students from diverse geographic and cultural backgrounds emerges as a tool for expanding access to quality education. The promotion of programs that encourage education and the mobility of Brazilian students, exemplified by the SWE/CNPq program, plays a significant role in ensuring equal opportunities and access to academic resources and advanced research infrastructure. In this regard, the sandwich PhD contributed to a deeper understanding of various global realities, thereby strengthening efforts to combat persistent social, economic, and educational inequalities across countries and regions.

During technical visits to local health services in Portugal, it was observed how non-material technologies, such as work management and organizational systems, played a crucial role in reorganizing patient care flows for those with respiratory symptoms and in ensuring the safety of frontline professionals during the pandemic. The availability of technological resources (electronic health records, computers, webcams, internet access), physical resources (individual and collective protective equipment, work instruments), and structural resources (ventilation and dimensions of health units, geographic distance from reference hospitals) was pivotal in confronting the pandemic, though unevenly distributed among countries. The availability, absence, and deaths of professionals presented a significant challenge worldwide, highlighting the scarcity of nursing professionals and its impact on response time and quality during new health crises.

The experience facilitated the sharing of knowledge and best practices in scientific research, as well as an understanding of the strengths and challenges in managing health systems in Brazil and Portugal. Although each country has its specificities, the adoption of comprehensive policies, such as fiscal, wage, and social protection measures, can contribute to addressing inequalities.

Regarding joint knowledge production, the sandwich PhD contributed to advances in studies related to worker health, safe and healthy work environments, and the appreciation of the health workforce. Furthermore, the production of scientific articles resulting from this research enabled the dissemination of findings in international academic forums, promoting global scientific cooperation. Overall, the experience provided the acquisition of new knowledge, the development of professional and teaching skills, and the integration of Brazilian PhD students into international research. The results indicate that the sandwich PhD plays a significant role in promoting international collaboration among universities, with a notable influence on achieving SDGs 4, 5,

### Challenges Encountered During the Overseas Fellowship and Strategies for Overcoming Them

Living in another country requires cultural adaptation, presenting both challenges and opportunities for generating new knowledge. Another difficulty is related to data collection, which demands significant time for institutional and ethics committee approval, as well as for sensitizing potential research participants. The time-related challenges were addressed with the involvement of the international supervisor, including the submission of materials to the ethics committee prior to the start of the fellowship.

## DISCUSSION

The concept of internationalization, while complex, is gaining increasing importance in fields such as science and higher education, particularly within the broader context of globalization and global education. Its relevance, however, extends beyond academia, with significant implications in sociopolitical and cultural spheres. Academic internationalization comprises “a comprehensive and dynamic process that encompasses education, research, and the provision of services to society”. Moreover, it serves as a tool to make higher education adaptable to the requirements and challenges of a globally interconnected society^(6)^.

In September 2023, representatives of the Member States gathered at the UN headquarters for the 78th General Assembly. The program included the opening of the SDG Summit and a keynote address by UN Secretary-General António Guterres, who reaffirmed the commitment made eight years ago when the SDGs were adopted, promising to “build a world of health, progress, and opportunities for all. A promise to leave no one behind”. Guterres also expressed concern that only 15% of the targets are on track to be met^(7)^.

The global revitalization of the SDGs marks the halfway point of the 2030 Agenda, initiated in 2015, and signals a new moment: it is essential to engage in this movement, embrace the cause, debate, and take action. During the 78th General Assembly, the 193 UN Member States adopted the Political Declaration of the SDG Summit, reaffirming their commitment to effectively implementing the 2030 Agenda and its SDGs, defending all its enshrined principles, and promoting systemic change toward a more inclusive, just, peaceful, resilient, and sustainable world for all^(8)^.

In the academic context, the SDGs are present in the themes of scientific events, and there is a growing interest in publications that relate them to various fields of knowledge, providing opportunities for the dissemination of science in alignment with the 2030 Agenda^(9, 10)^. The sandwich PhD plays a pivotal role in promoting the internationalization of academic research and strengthening global collaboration among universities, with the potential to reduce gender and educational inequalities, as indicated by SDGs 4, 5, and 10.

Given the global reality marked by pronounced socioeconomic, ethnic, gender, and social justice inequalities, it becomes imperative that nursing is taught, learned, and practiced from a global perspective, considering prevailing inequities. In this sense, international collaboration in the field of nursing promotes greater access to resources, increased scientific production, and the innovation of educational practices^(1)^.

The sandwich PhD contributes to the quality and diversity of research developed in Brazilian Graduate Programs and to building a truly global and interconnected academic community. By engaging in academic experiences in other countries, students enrich their training with intercultural perspectives and innovative approaches to teaching and research, in line with SDG 4. This immersion in diverse academic environments contributes to the development of critical skills, broadens access to specialized knowledge, and strengthens the commitment to educational excellence on a global scale^(2, 9)^. It is also worth highlighting the impact of these experiences on improving nursing practices in both Brazil and other countries around the world^(3)^.

By encouraging the participation of students from diverse genders and cultural backgrounds, the sandwich doctorate contributes to the creation of more equitable university environments where knowledge is constructed from multiple experiences^(2, 10)^. Academic mobility programs, including the sandwich doctorate, encourage students and researchers to develop their careers in an international context and lead scientific projects^(1, 2)^. These actions contribute to achieving SDG 5 by promoting gender equity in higher education institutions, fostering the distribution of financial resources for women-led research, and building a democratic space for the governance of education and institutions.

Furthermore, the sandwich PhD aligns with SDG 10 by facilitating access to learning and research opportunities in diverse international contexts. During the exchange, students have the chance to develop their skills at educational institutions abroad^(1, 2)^, regardless of age, gender, disability, race, ethnicity, origin, religion, economic status, or any other characteristic^(5)^.

SDGs 4, 5, and 10, focused on education, gender equality, and reducing inequalities, represent essential pillars for social and human progress. Through the sandwich PhD and international collaboration, significant advances can be made toward these objectives. In this context, overseas exchanges, knowledge sharing, and the development of joint actions contribute to a more equitable and sustainable world.

### Study limitations

This experience report, although based on the perspectives of those involved, offers insights that extend beyond the specific experience described. The reported experience provides a basis for broader reflections on the practice of the sandwich PhD.

### Contributions to the Field

In addition to encouraging other students to undertake a sandwich PhD, this experience report challenges academic institutions to build international interinstitutional partnerships aimed at strengthening the commitment made by countries to achieving the SDGs.

## FINAL CONSIDERATIONS

The exchange of knowledge, experiences, and cultural values among PhD students promotes sustainable development and the creation of a more inclusive global academic community committed to addressing contemporary global challenges. Through international collaboration facilitated by the sandwich PhD, it was possible to observe how promoting quality education and ensuring gender equality are fundamental to reducing inequalities and achieving sustainable development. This collaboration also strengthens nursing as a health profession with a highly relevant role in efforts to achieve the SDGs and highlights the need for joint actions and global efforts to meet the goals set by the 2030 Agenda.
