# Supplementary material for: Sandwich doctorate in Nursing: contributions to academic training and the Sustainable Development Goals
Source: Rev Bras Enferm. 2025 Jun 20;78(Suppl 1):e20240344. doi: 10.1590/0034-7167-2024-0344 (PMC12185112; doi:10.1590/0034-7167-2024-0344)
Supplement: Translation

# Translation: Doutorado sanduíche em Enfermagem: contribuições para a formação acadêmica e os Objetivos de Desenvolvimento Sustentável

**DOI:** 10.1590/0034-7167-2024-0344pt

**Authors:** Mariana Mendes, Denise Elvira Pires de Pires, Ianka Cristina Celuppi, Olga Maria Pimenta Lopes Ribeiro

**Affiliations:** I Universidade Federal de Santa Catarina. Florianópolis, Santa Catarina, Brasil; II Escola Superior de Enfermagem do Porto. Porto, Portugal

**Keywords:** Desenvolvimento Sustentável, Intercâmbio Educacional Internacional, Programas de Pós-Graduação em Saúde, Cooperação Técnica, Enfermagem

## Abstract

**Objetivos:**

relatar as contribuições do doutorado sanduíche para a formação de doutores em Enfermagem, com exploração e análise dos impactos na promoção da colaboração internacional entre programas de pós-graduação e sua contribuição para os Objetivos de Desenvolvimento Sustentável 4, 5 e 10.

**Métodos:**

relato de experiência de uma estudante de doutorado no exterior, realizado por meio de concessão de bolsa pelo Conselho Nacional de Desenvolvimento Científico e Tecnológico (CNPq), em uma Escola de Enfermagem de Portugal, entre maio e julho de 2023.

**Resultados:**

apresentados em três seções: atividades realizadas; contribuições do doutorado sanduíche para o alcance dos Objetivos 4, 5 e 10; dificuldades encontradas e estratégias para superação. O doutorado sanduíche promove a aquisição de novos conhecimentos, desenvolvimento profissional e para a docência, e parcerias internacionais.

**Considerações Finais:**

a colaboração internacional, facilitada pelo doutorado sanduíche, contribui para o fortalecimento da enfermagem como profissão de saúde, que tem papel altamente relevante no alcance das metas da Agenda 2030.

## INTRODUÇÃO

A colaboração internacional entre universidades tem se mostrado relevante para o avanço do conhecimento em Enfermagem e o fortalecimento das relações acadêmicas entre instituições de ensino em todo o mundo. Nesse contexto de colaboração, ressalta-se a internacionalização das ações entre universidades do Brasil e de Portugal, as quais têm contribuído para a mobilidade de discentes e docentes dos programas de pós-graduação, podendo ocorrer a partir de acordos oficiais de cooperação científica ou decorrentes de contatos em redes profissionais^(1)^.

As parcerias interinstitucionais internacionais possibilitam o intercâmbio acadêmico e a produção de conhecimento em diferentes contextos^(1, 2)^. O doutorado sanduíche no exterior refere-se a um modelo de formação acadêmica em que o estudante de doutorado realiza parte dos estudos relacionados à sua tese, a ser defendida no Brasil, em uma instituição de ensino estrangeira^(1, 3)^. Nessa perspectiva, o doutorado sanduíche no exterior se configura como uma ferramenta poderosa para impulsionar a colaboração internacional e a criação de novas redes de cooperação, além de contribuir para a formação de pesquisadores críticos e comprometidos com o desenvolvimento da ciência em nível global.

As experiências de cooperação internacional contribuem para uma melhor formação e desempenho profissional, especialmente ao oportunizar a troca de conhecimentos metodológicos e científicos, construindo caminhos para a prática baseada em evidências. Além disso, destacam-se as repercussões da vivência internacional na maturidade cultural, social e científica dos enfermeiros^(3)^, o que torna o doutorado sanduíche no exterior uma estratégia para desenvolver as habilidades e competências desses profissionais no âmbito internacional^(1)^.

O doutorado sanduíche é parte das estratégias de cooperação internacional. Por meio dele, os enfermeiros têm acesso a diferentes ambientes acadêmicos, recursos pedagógicos e tecnológicos, desenvolvem suas atividades em colaboração com pesquisadores internacionais e interagem com a cultura e sistemas locais. Além de contribuir para a formação profissional e acadêmica do pós-graduando^(1, 2)^, essas vivências ampliam as oportunidades de acesso à educação de qualidade e podem ser um recurso potente para reduzir as desigualdades sociais e de gênero.

O benefício dessa experiência se estende para além do desenvolvimento acadêmico e profissional do enfermeiro, contribuindo para a produção de diferentes formas de conhecimento, que impactam diretamente a coletividade^(1, 3)^, podendo assim colaborar para o alcance dos Objetivos de Desenvolvimento Sustentável (ODS). Em 2015, a Organização das Nações Unidas (ONU) aprovou 17 ODS, a serem alcançados até 2030. Esses objetivos consistem em diretrizes orientadoras de ações globais para reduzir a pobreza e as desigualdades entre grupos populacionais, incluindo a saúde, bem como para o investimento na paz e na preservação da vida no planeta.

Em julho de 2023, a ONU lançou a campanha “Aja Agora” pela defesa dos ODS, que antecedeu a 78ª Assembleia Geral das Nações Unidas, realizada em setembro do mesmo ano. Na campanha, o apelo ocorre em virtude da estagnação do desenvolvimento de ações para o alcance dos ODS, tendo em vista o “peso combinado de desastres climáticos, conflitos, recessão econômica e as consequências persistentes da COVID-19”^(4)^.

A realização do doutorado sanduíche permite que os estudantes adquiram uma perspectiva global e interajam com redes de pesquisa internacionais, para a criação de soluções para os problemas macropolíticos da sociedade, como está expresso nos ODS, incluindo a saúde. A experiência aqui relatada foi realizada em Portugal, como parte do processo de doutoramento de uma acadêmica brasileira. A pesquisa de doutoramento investiga inovações tecnológicas utilizadas por enfermeiros/as que atuaram na Atenção Primária de Saúde (APS) durante a pandemia de COVID-19, buscando entender seu processo de implementação e os desfechos no pós-pandemia, estudando o fenômeno em dois países, Portugal e Brasil.

Considerando a importância de impulsionar a excelência acadêmica no contexto da busca por soluções inovadoras e sustentáveis que contribuam para a consecução das metas previstas na Agenda 2030, formulou-se a seguinte pergunta norteadora deste estudo: Em que sentido a realização de estágio de doutorado sanduíche no exterior pode contribuir para avanços na formação de doutores e para o desenvolvimento sustentável?

O presente relato de experiência mostra as contribuições que o doutoramento sanduíche pode trazer para a formação e o desenvolvimento da ciência da Enfermagem, interpretando os resultados com inspiração nos esforços globais para a concretização de um mundo mais sustentável. Neste estudo, a busca pela consecução dos ODS é considerada, com foco especial em três deles: 4 - Educação de Qualidade, 5 - Igualdade de Gênero e 10 - Redução das Desigualdades^(5)^

## OBJETIVOS

Relatar as contribuições do doutorado sanduíche para a formação de doutores em Enfermagem, com exploração e análise dos impactos na promoção da colaboração internacional entre programas de pós-graduação e sua contribuição para os ODS 4, 5 e 10.

## MÉTODOS

### Tipo de estudo

Este estudo adota uma abordagem qualitativa, descritiva, caracterizando-se como um relato de experiência, com o objetivo de evidenciar as contribuições do doutorado sanduíche no exterior para a formação de doutores em Enfermagem e para os ODS 4, 5 e 10.

### Alicerces teóricos

Para a conceitualização e debate acerca dos ODS, utilizou-se o disposto no site oficial da ONU Brasil^(5)^ e consulta intencional a documentos oficiais, relatórios institucionais e publicações científicas sobre o tema disponíveis na internet.

### Período e contexto do estudo

A experiência ocorreu durante a realização de um doutoramento sanduíche no exterior por uma aluna brasileira, vinculada a um Programa de Pós-Graduação em Enfermagem de uma Universidade Federal, na região Sul do Brasil. O cenário da experiência foi uma Escola de Enfermagem em Portugal. Envolveu a concessão de bolsas de doutorado sanduíche no exterior (SWE) pelo Conselho Nacional de Desenvolvimento Científico e Tecnológico (CNPq), em resultado da submissão de propostas à Chamada CNPq Nº 26/2021 de Apoio à Pesquisa Científica, Tecnológica e de Inovação - Bolsa no Exterior. A bolsa teve duração de três meses, realizada entre maio e julho de 2023.

O aceite da aluna na instituição no exterior foi formalizado mediante contato entre as professoras do Brasil e de Portugal, oportunizado pela relação de colaboração internacional pré-existente entre ambas. O contato prévio foi fundamental para a elaboração do projeto de pesquisa, contendo a proposta de estudo, incluindo o planejamento e o cronograma das atividades a serem realizadas no âmbito das atividades da pós-graduação da instituição de destino no exterior.

Organização dos dados: Os dados foram organizados em seções temáticas para atender aos objetivos do estudo. Essas seções incluem: a descrição das atividades realizadas no exterior e sua relação com a formação de doutores, a produção de conhecimento em Enfermagem e a contribuição para os ODS; a caracterização dos ODS 4, 5 e 10 e sua associação com a experiência do doutorado sanduíche; e a discussão das dificuldades enfrentadas e das estratégias de superação.

### Aspectos éticos

Por se tratar de um relato de experiência, o estudo não necessitou de aprovação do Comitê de Ética em Pesquisa com Seres Humanos. No entanto, respeitou-se o preconizado nos preceitos internacionais e nas Resoluções 466/12 e 510/16 do Conselho Nacional de Saúde, Brasil, preservando o anonimato e a confidencialidade para fins de pesquisa.

## RESULTADOS

Os resultados da experiência estão apresentados em três seções: atividades realizadas; contribuições do doutorado sanduíche para o alcance dos ODS 4, 5 e 10; dificuldades encontradas e estratégias para superação.

### Atividades realizadas durante o estágio de doutorado sanduíche no exterior

O Quadro 1 apresenta as atividades realizadas durante o estágio de doutorado sanduíche, entre maio e julho de 2023, relacionando-as com as contribuições para a formação doutoral e o avanço da ciência, bem como para o alcance dos ODS.

**Quadro 1:** Atividades realizadas, contribuições para a formação doutoral, para a ciência e parcerias em pesquisa, e para o alcance dos Objetivos de Desenvolvimento Sustentável

Atividades realizadas	Contribuições para a formação doutoral, para a ciência e parcerias em pesquisa	Relações com o/os ODS
Palestrante no evento *NursID Spring School,* compartilhando pesquisas realizadas no Brasil acerca de inovações tecnológicas na saúde e relações com a saúde do trabalhador, assim como, perspectivas teóricas do projeto de doutorado.	Divulgação do conhecimento e intercâmbio científico.	ODS 4 e 5
Participação em aulas e eventos acadêmicos e científicos do Brasil e internacionais, nas modalidades on-line e presencial.	Divulgação de pesquisas acerca de tecnologias desenvolvidas e utilizadas pela enfermagem no Brasil.	ODS 4 e 10
Visitas técnicas em unidades de saúde locais e hospital referência no atendimento a COVID-19.	Identificação de formas de organização e gestão dos serviços de saúde e, também, do processo de trabalho do enfermeiro, com possíveis aplicações em outros cenários.	ODS 10
Coleta de dados com enfermeiros da APS acerca das tecnologias utilizadas na pandemia de COVID-19.	Enriquece a produção de conhecimentos, ampliando a compreensão do fenômeno, Brasil-Portugal.	ODS 4 e 5
Participação nas atividades dos grupos de pesquisa *Positive Professional Environments for Nursing* Practice-Portugal, e do Laboratório de Pesquisa sobre Trabalho, Ética, Saúde e Enfermagem (PRAXIS) -Brasil.	Intercâmbio e fortalecimento de redes de pesquisa.	ODS 4 e 10
Colaboração na produção de artigos científicos, elaborados em parceria com pesquisadores internacionais.	Divulgação dos resultados de pesquisas científicas conduzidas por mulheres e do conhecimento em enfermagem.	ODS 4 e 5

### Contribuições para o alcance dos Objetivos de Desenvolvimento Sustentável

A Figura 1 mostra os ODS selecionados, 4, 5 e 10 e relações com três temas articuladores dos contributos do doutorado sanduíche para o seu alcance.

**Figura 1:** Objetivos de Desenvolvimento Sustentável e a experiência do doutorado sanduíche no exterior

### Internacionalização da pesquisa acadêmica e a colaboração internacional entre universidades

A troca de conhecimentos entre pesquisadores de diferentes nacionalidades impulsiona a qualidade e a inovação das pesquisas. O doutorado sanduíche permite a partilha de abordagens distintas no âmbito da pesquisa, essenciais para enfrentar os desafios complexos que a sociedade enfrenta atualmente. Dessa forma, o doutorado sanduíche, além de promover a internacionalização da pesquisa acadêmica e fortalecer a colaboração global entre universidades, potencializa o desenvolvimento e a expertise dos profissionais/pesquisadores envolvidos, alinhando-se ao ODS 4, que trata da educação de qualidade em todo o mundo.

A participação em eventos acadêmicos e científicos no exterior proporcionou a divulgação de pesquisas realizadas por enfermeiros brasileiros e o diálogo com pesquisadores internacionais. Também possibilitou aproximações com diferentes metodologias de pesquisa e com o desenvolvimento de estudos clínicos e de tecnologias aplicadas ao trabalho dos enfermeiros portugueses (programas de reabilitação para pacientes cirúrgicos, tecnologia para avaliação do equilíbrio e marcha em idosos). Essa vivência internacional inspira o desenvolvimento de pesquisas mais robustas, com vistas a melhorias no campo da saúde e enfermagem.

Um aspecto particularmente valioso da experiência é a oportunidade de coletar dados para a tese em um contexto internacional. Os dados coletados no Brasil e em Portugal contribuem para a produção de uma tese mais consistente e com maior relevância global, favorecendo o debate crítico acerca da implementação de tecnologias, em especial pelos enfermeiros da APS. Por meio de entrevistas com os profissionais participantes, visitas técnicas e interações com os serviços de saúde locais, foi possível conhecer experiências exitosas dos enfermeiros na utilização de tecnologias inovadoras durante a pandemia, com destaque para as plataformas de videoconferência para comunicação síncrona com os usuários e o telemonitoramento de pacientes com a doença.

O doutorado sanduíche também proporciona a oportunidade de estabelecer relações de parceria e cooperação com pesquisadores líderes em suas áreas de estudo. A participação da doutoranda em um grupo de pesquisa internacional, dedicado à investigação de ambientes positivos para a prática da enfermagem, ampliou o conhecimento acerca das tecnologias utilizadas e suas repercussões na assistência à saúde e na saúde do trabalhador.

O estágio sanduíche impulsionou o desenvolvimento de competências na produção e submissão de artigos científicos em periódicos internacionais, exigindo a compreensão de diferentes idiomas, estilos de escrita e formatação dos trabalhos acadêmicos. O desenvolvimento dessas habilidades acadêmicas também é útil na construção de redes e futuras parcerias internacionais.

### Empoderamento feminino e igualdade de gênero na academia

O doutorado sanduíche desempenha um papel crucial na promoção da igualdade de gênero no contexto acadêmico. Isso se reflete na capacidade de oferecer igualdade de oportunidades às mulheres estudantes e pesquisadoras, permitindo que acessem ambientes acadêmicos internacionais, contribuam com suas

pesquisas e estabeleçam redes de contatos que fortalecem seu empoderamento e influenciam positivamente a representação de gênero na pesquisa e no ensino superior. Esse processo contribui para a realização do ODS 5, que visa eliminar desigualdades de gênero e promover a participação igualitária das mulheres em todos os setores da sociedade.

A vivência da doutoranda reforçou o papel fundamental das mulheres no desenvolvimento da ciência da Enfermagem e na construção do conhecimento acadêmico, especialmente em uma profissão predominantemente feminina. A condução de estudos clínicos, o desenvolvimento de tecnologias inovadoras, a coordenação de projetos de pesquisa e a docência na pós-graduação são exemplos de atividades lideradas por professoras envolvidas na orientação deste estágio. A presença de mulheres em cargos de alta gestão, como as duas vice-presidentes da Escola, também evidencia as possibilidades de participação e oportunidades de liderança.

Por outro lado, a vivência permitiu a troca de experiências sobre os desafios enfrentados por mulheres que desejam se desenvolver na área acadêmica, como a gestão das múltiplas dimensões da vida da mulher, que compõem a tríade mãe/esposa/profissional. Essa vivência estimulou reflexões sobre os múltiplos papéis atribuídos às mulheres na sociedade e como eles podem influenciar as desigualdades de oportunidades no mercado e na vida acadêmica.

### Acesso equitativo à educação e redução das desigualdades

No contexto do ODS 10, o intercâmbio entre estudantes de doutorado de diversas origens geográficas e culturais emerge como uma ferramenta para a ampliação do acesso à educação de qualidade. A promoção de programas de incentivo à educação e à mobilidade de estudantes brasileiros, exemplificada pelo programa SWE/CNPq, desempenha um papel significativo na garantia da igualdade de oportunidades e no acesso a recursos acadêmicos e à infraestrutura avançada de pesquisa. Nesse sentido, o doutorado sanduíche contribuiu para uma compreensão mais aprofundada das diversas realidades globais, fortalecendo, assim, os esforços para combater as desigualdades sociais, econômicas e educacionais que ainda persistem entre países e regiões.

Durante as visitas técnicas aos serviços de saúde locais em Portugal, observou-se como as tecnologias não materiais, como as de gestão e organização do trabalho, desempenharam um papel crucial na reorganização dos fluxos de atendimento de pacientes com sintomas respiratórios e na segurança dos profissionais na linha de frente durante a pandemia. A disponibilidade de recursos tecnológicos (prontuário eletrônico, computadores, *webcams,* acesso à internet), físicos (equipamentos de proteção individual e coletiva, instrumentos de trabalho) e estruturais (ventilação e dimensões das unidades de saúde, distância geográfica dos centros hospitalares de referência) foi determinante para o enfrentamento da pandemia, embora se apresente de forma desigual entre os países. A disponibilidade, afastamento e óbitos de profissionais constituíram-se em um grande desafio em todo o mundo, alertando para a escassez de profissionais de enfermagem, o que repercutirá no tempo e na qualidade da resposta em novas crises sanitárias.

A vivência possibilitou o compartilhamento de conhecimentos e boas práticas na pesquisa científica, bem como a compreensão das potencialidades e desafios na gestão dos sistemas de saúde no Brasil e em Portugal. Embora cada país tenha suas especificidades, a adoção de políticas abrangentes, como as fiscais, salariais e de proteção social, pode contribuir para o enfrentamento das desigualdades.

No que diz respeito à produção conjunta do conhecimento, o doutorado sanduíche contribuiu para o avanço em estudos relacionados à saúde dos trabalhadores, ambientes de trabalho seguros e saudáveis e à valorização da força de trabalho na área da saúde. Além disso, a produção de artigos científicos resultantes dessas pesquisas possibilitou a divulgação dos resultados em fóruns acadêmicos internacionais, promovendo a cooperação científica global. De modo geral, a experiência proporcionou a aquisição de novos conhecimentos, o desenvolvimento de competências profissionais e para a docência, bem como a inserção de doutorandos brasileiros na pesquisa internacional. Os resultados indicam que o doutorado sanduíche possui um papel relevante na promoção da colaboração internacional entre universidades, com influências para a consecução dos ODS 4, 5 e 10.

### Dificuldades encontradas no estágio no exterior e estratégias para superação

A vida em outro país demanda adaptação cultural, representando, ao mesmo tempo, um desafio e uma oportunidade para a geração de novos conhecimentos. Outra dificuldade diz respeito à coleta de dados, que envolve um tempo significativo para aprovação institucional e pelo comitê de ética, bem como para a sensibilização dos potenciais sujeitos participantes da pesquisa. As dificuldades relacionadas ao tempo foram superadas com o envolvimento da supervisora internacional, incluindo o envio do material ao comitê de ética antes do início do estágio.

## DISCUSSÃO

O conceito de internacionalização, embora complexo, ganha cada vez mais importância em campos como a ciência e o ensino superior, especialmente dentro do cenário mais amplo de globalização e educação global. Sua relevância, contudo, se estende além da academia, com implicações significativas nas esferas sociopolíticas e culturais. A internacionalização acadêmica compreende “um processo abrangente e dinâmico que engloba educação, pesquisa e provisão de serviços para a sociedade”. Além disso, serve como uma ferramenta para tornar a educação superior adaptativa aos requisitos e desafios de uma sociedade globalmente interligada^(6)^.

Em setembro de 2023, os representantes dos Estados-membros reuniram-se na sede da ONU para a 78^a^ Assembleia Geral. A programação incluiu a abertura da Cúpula dos ODS e um discurso auspicioso do secretário-geral da ONU, António Guterres, que reafirmou o compromisso assumido há 8 anos, quando adotaram os ODS e prometeram “construir um mundo de saúde, progresso e oportunidades para todas as pessoas. Uma promessa de não deixar ninguém para trás”. Guterres também expressou sua preocupação com o fato de que apenas 15% das metas estão no caminho para serem cumpridas^(7)^.

O resgate global acerca dos ODS marca a metade do prazo para a concretização da Agenda 2030, iniciado em 2015, e sinaliza para um novo momento: é preciso participar desse movimento, aderir à causa, debater e agir. Durante a 78a Assembleia Geral, os 193 Estados-membros da ONU adotaram a Declaração Política da Cúpula dos ODS, na qual reafirmam o compromisso de implementar eficazmente a Agenda 2030 e os seus ODS, defender todos os princípios nela consagrados e promover uma mudança sistêmica em direção a um mundo mais inclusivo, justo, pacífico, resiliente e sustentável para todos^(8)^.

No contexto acadêmico, os ODS estão presentes em temáticas de eventos científicos, e visualiza-se um maior interesse em publicações que os relacionem às diversas áreas do conhecimento, oportunizando a divulgação da ciência em consonância com a Agenda 2030^(9, 10)^. O doutorado sanduíche desempenha um papel articulador na promoção da internacionalização da pesquisa acadêmica e no fortalecimento da colaboração global entre universidades, com potencial para reduzir as desigualdades de gênero e educacionais, como indicam os ODS 4, 5 e 10.

Dada a realidade global marcada por notórias desigualdades socioeconómicas, étnicas, de gênero e de justiça social, torna-se imprescindível que a enfermagem seja ensinada, aprendida e exercida em uma perspectiva global, considerando as iniquidades prevalecentes. Nesse sentido, a colaboração internacional na área de enfermagem promove maior acesso a recursos, aumento da produção científica e inovação das práticas educativas^(1)^.

O estágio de doutorado sanduíche contribui para a qualidade e diversidade das pesquisas desenvolvidas nos Programas de Pós-Graduação do Brasil, assim como para a construção de uma comunidade acadêmica verdadeiramente global e interconectada. Ao vivenciar experiências acadêmicas em outros países, os estudantes enriquecem sua formação com perspectivas interculturais e abordagens inovadoras de ensino e pesquisa, em consonância com o ODS 4. Essa imersão em ambientes acadêmicos diversos contribui para o desenvolvimento de habilidades críticas, amplia o acesso a conhecimentos especializados e fortalece o compromisso com a excelência educacional em âmbito global^(2, 9)^. Cabe também destacar o impacto dessas vivências na melhoria das práticas de enfermagem, tanto no Brasil quanto em outros países do mundo^(3)^.

Ao incentivar a participação de estudantes de diferentes gêneros e origens culturais, o doutorado sanduíche contribui para a construção de ambientes universitários mais justos, onde o conhecimento é construído a partir das múltiplas experiências^(2, 10)^. Os programas de mobilidade acadêmica, incluindo o doutorado sanduíche, incentivam que estudantes e pesquisadoras desenvolvam suas carreiras no contexto internacional e liderem projetos científicos^(1, 2)^. Essas ações contribuem para o alcance do ODS 5, promovendo a equidade de gênero em instituições de ensino superior, fomentando a distribuição de recursos financeiros para pesquisas lideradas por mulheres e a construção de um espaço democrático na gestão do ensino e das instituições.

Além disso, o doutorado sanduíche está alinhado ao ODS 10, ao facilitar o acesso a oportunidades de aprendizado e pesquisa em diferentes contextos internacionais. Durante o intercâmbio, os estudantes têm a chance de desenvolver suas habilidades em instituições de ensino no exterior^(1, 2)^, independentemente de idade, gênero, deficiência, raça, etnia, origem, religião, condição econômica ou qualquer outra característica^(5)^.

Os ODS 4, 5 e 10, focados na educação, igualdade de gênero e redução das desigualdades, representam pilares essenciais para o progresso social e humano. Por meio do doutorado sanduíche e da colaboração internacional, é possível impulsionar avanços significativos em direção a esses objetivos. Nesse contexto, o intercâmbio no exterior, a troca de conhecimentos e o desenvolvimento de ações conjuntas contribuem para um mundo mais igualitário e sustentável.

### Limitações do estudo

Este relato de experiência, embora baseado na perspectiva dos envolvidos, oferece insights que vão além da experiência específica descrita, fornecendo uma base para reflexões mais amplas sobre a prática do doutorado sanduíche.

### Contribuições para a Área

Este relato de experiência, além de desafiar outros estudantes a realizar o doutorado sanduíche, incita as instituições acadêmicas à construção de parcerias interinstitucionais internacionais, com vistas a potencializar o compromisso assumido pelos países para a concretização dos ODS.

## CONSIDERAÇÕES FINAIS

A troca de conhecimentos, experiências e valores culturais entre estudantes de doutorado promove o desenvolvimento sustentável e a construção de uma comunidade acadêmica global mais inclusiva e comprometida com os desafios globais da contemporaneidade.

Por meio da colaboração internacional facilitada pelo doutorado sanduíche, foi possível perceber como a promoção da educação de qualidade e a garantia da igualdade de gênero são fundamentais para a redução das desigualdades e para o alcance de um desenvolvimento sustentável. Essa colaboração também fortalece a enfermagem como uma profissão da saúde que tem papel altamente relevante nos esforços para o alcance dos ODS e evidencia a necessidade de ações conjuntas e esforços globais para alcançar os objetivos propostos pela Agenda 2030.
